# Limited spatial response to direct predation risk by African herbivores following predator reintroduction

**DOI:** 10.1002/ece3.2312

**Published:** 2016-07-22

**Authors:** Andrew B. Davies, Craig J. Tambling, Graham I.H. Kerley, Gregory P. Asner

**Affiliations:** ^1^ Department of Global EcologyCarnegie Institution for Science260 Panama StreetStanfordCalifornia94305; ^2^ Department of ZoologyCentre for African Conservation EcologyNelson Mandela Metropolitan UniversityPort Elizabeth6031South Africa

**Keywords:** Antipredator behavior, camera traps, Carnegie Airborne Observatory, habitat selection, landscape of fear, Light Detection and Ranging, lion, *Panthera leo*, predator reintroductions, predator–prey interactions, thicket biome

## Abstract

Predators affect ecosystems not only through direct mortality of prey, but also through risk effects on prey behavior, which can exert strong influences on ecosystem function and prey fitness. However, how functionally different prey species respond to predation risk and how prey strategies vary across ecosystems and in response to predator reintroduction are poorly understood. We investigated the spatial distributions of six African herbivores varying in foraging strategy and body size in response to environmental factors and direct predation risk by recently reintroduced lions in the thicket biome of the Addo Elephant National Park, South Africa, using camera trap surveys, GPS telemetry, kill site locations and Light Detection and Ranging. Spatial distributions of all species, apart from buffalo, were driven primarily by environmental factors, with limited responses to direct predation risk. Responses to predation risk were instead indirect, with species distributions driven by environmental factors, and diel patterns being particularly pronounced. Grazers were more responsive to the measured variables than browsers, with more observations in open areas. Terrain ruggedness was a stronger predictor of browser distributions than was vegetation density. Buffalo was the only species to respond to predator encounter risk, avoiding areas with higher lion utilization. Buffalo therefore behaved in similar ways to when lions were absent from the study area. Our results suggest that direct predation risk effects are relatively weak when predator densities are low and the time since reintroduction is short and emphasize the need for robust, long‐term monitoring of predator reintroductions to place such events in the broader context of predation risk effects.

## Introduction

Predators alter ecosystems by influencing prey species via both direct killing and indirect risk‐related processes (Lima 1998; Ripple et al. 2014b). Direct mortality can lead to declines in prey populations (Eberhardt et al. 2007) and is the most commonly measured consequence of predation (Creel and Christianson 2008; Creel 2011). However, a growing number of studies have demonstrated that risk effects can be as strong as, or even stronger, than consumptive effects (Preisser et al. 2005; Pangle et al. 2007; Creel and Christianson 2008; but see Middleton et al. 2013), sometimes leading to trophic cascades and alterations in the structure and functioning of ecosystems (Ford et al. 2014; Ripple et al. 2014a).

Predation risk is often defined as the probability of prey encountering a predator (encounter risk) and/or being killed (mortality risk) in a particular location (e.g., Valeix et al. 2009b; Thaker et al. 2011). Both of these elements of predation risk affect prey behavior because neither are homogenous across landscapes, nor are they random. In contrast, both vary in space and time and can often be predicted by prey. Prey respond to such variation in risk through adjustments to their behavior, including changes in group size (Tambling et al. 2012; Creel et al. 2014), vigilance (Périquet et al. 2012; Creel et al. 2014), movement patterns (Fortin et al. 2005; Basille et al. 2015), timing of activity (Valeix et al. 2009a; Tambling et al. 2015), and spatial distributions (Creel et al. 2005; Valeix et al. 2009b; Courbin et al. 2016). Many of these adjustments can come at a cost to individual and population fitness because of a decrease in foraging time and/or because prey species are forced to forage in habitats with suboptimal forage availability, potentially reducing reproductive rates and affecting prey demography (Creel et al. 2007). Although smaller‐scale experimental studies have demonstrated strong and widespread effects of predation risk (e.g., Preisser et al. 2007; Schmitz 2008; Miller et al. 2014), less is known about risk effects at larger landscape levels, especially in relation to vertebrates. Moreover, studies that have investigated risk effects in relation to vertebrates have largely considered single predator–prey dyads in temperate regions (e.g., Fortin et al. 2005; Winnie and Creel 2007; Nicholson et al. 2014). This has limited our understanding of risk effects and our ability to predict their consequences and costs across different prey species and ecosystems, with little known about how characteristics of predators, prey, and/or the environment influence responses (Creel 2011).

The strength of risk effects can vary with attributes of both the predator and prey species. Hunting mode of the predator is one such determinant, with ambush predators exerting stronger effects than active searching predators, a pattern consistent across ecosystems, and prey functional groups (Schmitz 2008; Thaker et al. 2011; Miller et al. 2014). However, differences in prey traits are expected to mediate antipredator responses even more than predator hunting mode because prey characteristics affect all aspects of prey response to risk, compared with predator traits that affect only some responses (Creel 2011). Although little is known about the factors driving variation in antipredator responses within and between prey species, foraging strategy and body size have been shown to mediate response strength. In African savannas, browsers displayed stronger responses to predator encounter risk than grazers (Valeix et al. 2009b), and changes in vigilance and grouping behavior varied across prey species of different body sizes (Périquet et al. 2012; Creel et al. 2014). These studies suggest that prey characteristics, in conjunction with predator hunting mode, could be used to develop general predictions about antipredator behavior.

The role of the environment is also likely to be important in determining the strength of antipredator responses. Landscape heterogeneity has a strong influence on where predators make kills (mortality risk) (Hopcraft et al. 2005; Kauffman et al. 2007), and prey species can therefore be expected to alter their response to both mortality and predator encounter risk with environmental variation across landscapes. Concurrently, prey species can make use of the environment to avoid detection and/or escape predators (Creel et al. 2005), and as such, prey sometimes select environments known to have more predators, but to provide better means of escape and are therefore less risky than areas with higher predator encounter risk (Heithaus et al. 2009; Wirsing et al. 2010). Furthermore, foraging requirements can constrain herbivores to areas with sufficient forage, resulting in spatial distributions being determined largely by environmental variables rather than predation risk when forage is limiting (Sinclair 1985; Valeix et al. 2009b; Hopcraft et al. 2010). Moreover, where the ratio of predator to prey density is particularly low, predator regulation of prey populations is weak, and bottom‐up mechanisms are more important (Vucetich et al. 2011), with the strength of risk effects likely being concomitantly weak. Diel patterns can also influence antipredator responses, with prey species adjusting their behavior according to when predators are less active (Valeix et al. 2009a; Tambling et al. 2015). Responses to environmental variables and perceived predation risk are therefore likely to vary temporally between day and night.

Predator–prey dynamics are also changing in many ecosystems, with predator (and prey) extirpations in many areas (Ripple et al. 2014b, 2015) contrasted with reintroductions elsewhere (Hayward and Somers 2009). Although such reintroductions advance conservation efforts and can lead to improved ecosystem integrity and the restoration of ecological processes (Ripple and Beschta 2012), the landscapes into which predators are returning are often vastly different, smaller, and fragmented compared to their original state, as are the new predator populations. However, such isolated areas are rapidly becoming the last strongholds for many species (Ceballos et al. 2005; Packer et al. 2013), and so there is a need for improved understanding of predator–prey dynamics within them.

**Figure 1:**
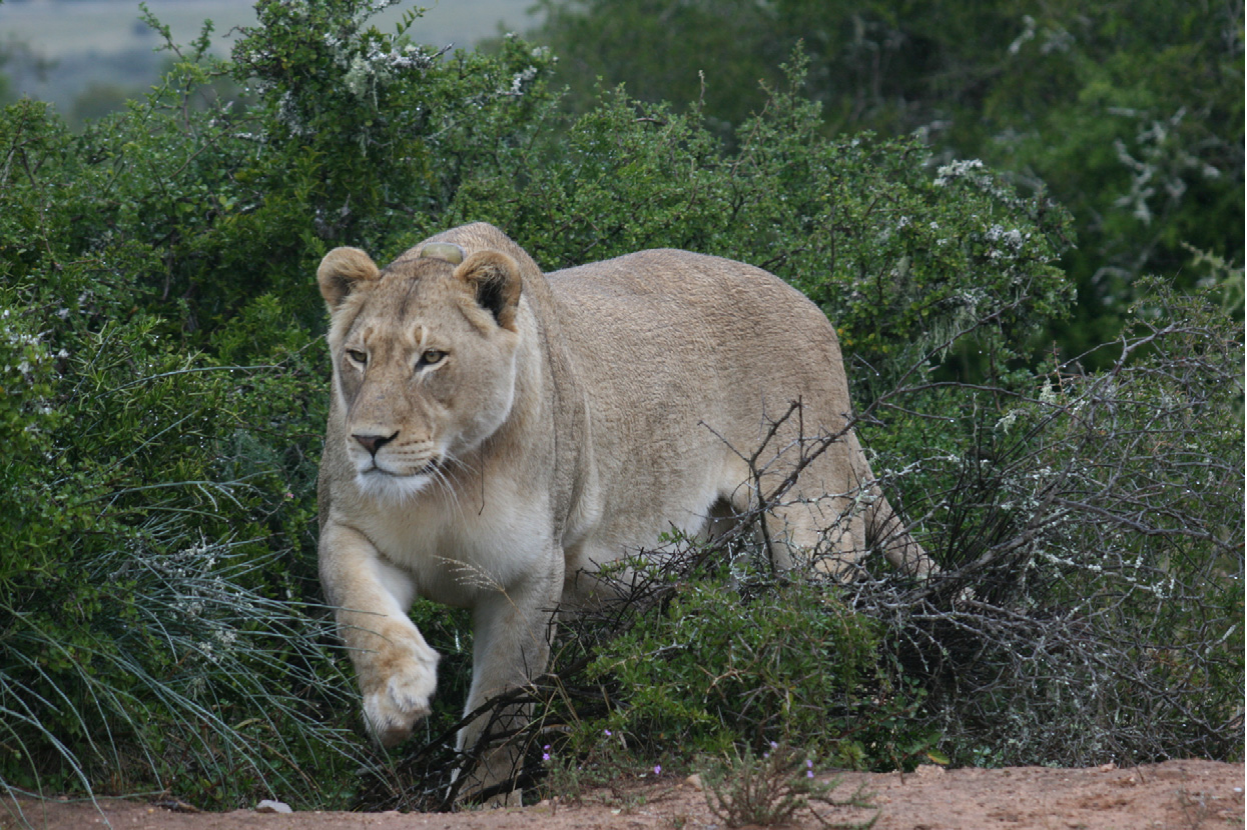
A lioness collared with a GPS satellite collar in Nyathi, Addo Elephant National Park, South Africa. The vegetation in the background is typical of the dense succulent thicket dominating much of the study site.

Here, we use a combination of GPS telemetry, kill site data, camera trap surveys, and airborne Light Detection and Ranging (LiDAR) to investigate effects of predation risk by recently reintroduced lions (*Panthera leo*) (Fig. 1) on the relative habitat‐specific abundance of six African herbivores varying in body size and foraging strategy in South Africa's subtropical thicket biome. We tested (1) whether habitat characteristics or direct predation risk factors were more influential in determining species' spatial distributions, (2) whether habitat characteristics, direct predation risk, and diel patterns interact to alter herbivore distributions, and (3) whether spatial distribution patterns differ between herbivore species varying in body size and foraging strategy. We expected herbivores to display differential habitat preferences based on their foraging strategy (browsers being found in areas of dense vegetation compared to grazers) and the strength of predation risk effects to vary with both herbivore body size (larger species being less susceptible), foraging strategy [with grazers being less able to adjust their distributions in response to predation risk, following Valeix et al. (2009b)] and time of day (with all species being less active at night when lions actively hunt).

## Methods

### Study site

**Figure 2:**
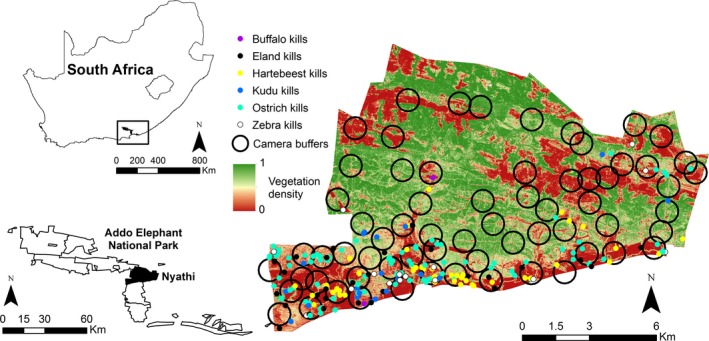
Map of the Nyathi section of the Addo Elephant National Park, South Africa, showing the position of the 500 m buffers around each of the 63 camera trap sites, the locations of the kills for each herbivore species and vegetation density across the landscape.

The study was conducted in the 140 km^2^ Nyathi section of the Addo Elephant National Park, South Africa (33°23′S, 25°50′E) (Fig. 2). Nyathi is entirely fenced and consists of open plains in the south (remnants of previous agricultural activity) and parts of the more rugged Zuurberg Mountains in the north. The vegetation is dominated by succulent thicket, which is typically evergreen and dense, reaching between 3 and 5 m tall (Kerley and Landman 2006). The region is semi‐arid with rainfall occurring throughout the year; mean annual precipitation is between 260 and 530 mm. Lions (Fig. 1) were reintroduced to Nyathi in September 2011, following an absence of over 100 years. Three lions in two social groups (a solitary lioness and two males in a coalition) were present during the study (2.1 lions/100 km^2^). No other large predators are common in Nyathi, although leopards (*Panthera pardus*) are known to occur in very low numbers, likely being nonresident. We collected a total of six leopard photographs, each with different camera traps, compared with 45 for lions during the study. We investigated habitat preference and possible effects of lion predation risk on six medium to large herbivore species that vary in foraging strategy and body size and are relatively common in Nyathi. These included one strict browser (kudu, *Tragelaphus strepsiceros*), a mixed feeder (eland, *Tragelaphus oryx*), and four grazers (buffalo, *Syncerus caffer*; plains zebra, *Equus quagga*; red hartebeest, *Alcelaphus buselaphus;* and ostrich, *Struthio camelus*). We used published body masses of each species (Davies and Bertram 2003 for ostrich, Smith et al. 2003 for mammals) to classify species according to body size, with buffalo and eland classified as large‐bodied (>500 kg), zebra, kudu, and hartebeest as medium‐bodied (>150, <300 kg), and ostrich as small‐bodied (<100 kg).

### Camera trap surveys

Camera traps (ScoutGuard SG550, Norcross, GA, Bushnell Trophy Cam XLT, Overland Park, KS and UWAY Vigilant Hunter U150, Norcross, GA) were deployed at 63 sites across Nyathi between December 2012 and August 2015 (Fig. 2). The study area was overlaid with a 1 × 1 km grid (approximately 150 grid cells). Forty‐one of these cells were inaccessible due to dense vegetation and no access tracks, leaving a total of 109 grid cells available for sampling. Due to logistical and camera constraints, we were able to sample 63 of these grid cells and therefore 42% of the study site. A single camera trap was placed in each grid cell, with cameras primarily located along animal paths and in close proximity to roads to facilitate maintenance. On average, sampling sites were spaced approximately 1 km apart, but temporally separated and rotated so that each broad region of the study site was sampled simultaneously. At any one time, the average distance between cameras was approximately 5 km. This sampling design was applied in an attempt to ensure wide coverage of the entire study site with the limited number of available cameras. Cameras were set at approximately 0.5–1.0 m above ground level, enabling the capture of images of a wide range of small and large mammals, set on low sensitivity and programmed to take two consecutive pictures with a quiescent time of 5 sec. Cameras were active at each site for a minimum of 90 days, resulting in an average of 103 (standard deviation of 20.2) days per camera. Deployed cameras that were active for <30 days were excluded from analysis. Cameras were checked roughly once a month to download photographs and to perform maintenance. Cameras were moved between sites during the course of the study with an average of 6.75 (SD = 3.1) cameras active at any one time.

Camera trap photographs were sorted according to species, with 30 min used as the time to independence between images (Linkie and Ridout 2011; Tambling et al. 2015). This time to independence assumes that multiple photographs of a species taken at a single site within a short period of time represent a single capture event and thus a single independent record. Thirty‐minute intervals were considered a fair compromise between the likelihood of capturing the same group multiple times and the likelihood of missing groups. Photographs separated by the time to independence (30 min) were assumed to be a representative random sample of the large animal population at each site. Observations were considered as a group of each species, rather than individuals of each species that could be counted in the images. Photographs were then classified as being taken during the day or night based on the local sunrise and sunset times over the course of the year, extracted using the function “crepuscule” in the R package maptools (Bivand and Lewin‐Koh 2016).

### Light Detection and Ranging data

We mapped the full extent of Nyathi with discrete‐return airborne LiDAR in March 2014 using the Carnegie Airborne Observatory (CAO, Asner et al. 2012). The CAO LiDAR subsystem provides three‐dimensional structural information of vegetation canopies and the underlying terrain surface. The GPS‐IMU subsystem provides three‐dimensional position and orientation data for the CAO sensors, allowing for highly precise and accurate projection of LiDAR observations on the ground. For this study, the CAO data were collected from 2000 m above ground level, using a scan angle of 36° and a side overlap of 50%, providing maps of ground elevation, woody canopy height, and three‐dimensional structure at 1.0 m spatial resolution. LiDAR measurements of vegetation height were field‐validated in early July 2014, and linear regression indicated a strong positive relationship between vegetation height measured in the field and with the LiDAR (*r* ^2^ = 0.90, *P *<* *0.001). Horizontal and vertical error estimates were 16 and 7 cm root mean square error (RMSE), respectively. Although the vegetation structure may have changed over the course of the 17 months preceding and/or after the LiDAR data collection, we assumed that because perennial woody biomass accounts for the majority of thicket vegetation, the general structure of the vegetation would be largely unchanged over this time scale. Furthermore, because thicket vegetation is evergreen, dominated by perennials and rainfall occurs throughout the year in Addo (Kerley and Landman 2006), seasonal differences in vegetation structure will be negligible.

Digital elevation models (DEMs) were fitted to the LiDAR point cloud data to estimate ground and top‐of‐canopy surfaces. Canopy height was calculated as the difference between the ground and canopy DEM. The ground DEM was resampled with bilinear interpolation to a 10 m cell size, and from this we calculated slope and terrain ruggedness (vector ruggedness measure, VRM, Sappington et al. 2007) using a 3 × 3 neighborhood. Similarly, vegetation density was measured as the proportion of vegetation cover above a height of 1 m (vegetation below this height was considered less relevant to predation risk) in each 10 by 10 m cell from a 10 m top‐of‐canopy DEM. Interpolated mean values for slope, VRM, and vegetation density within buffers around each camera location (*n* = 63) were then extracted and used to characterize each site. A buffer size of 500 m was constructed around each camera trap to characterize the general environment, while reducing overlap between cameras (Fig. 2).

### Lion telemetry and kill sites

GPS telemetry and kill data for the lions were collected between September 2011 and June 2014. A single female and two male lions (the latter being related and in a coalition) were collared by South African National Parks with GPS collars (GPS/GSM and GPS/Satellite units, African Wildlife Tracking, Pretoria, South Africa) when released into Nyathi and set to download between 3 and 5 GPS locations per day (periodically some collars were programmed to record more locations per day). This encompassed all the lions present in Nyathi at the time of the study, allowing an accurate assessment of lion ranging and killing behavior, and thus predation risk. Lion collaring was conducted in accordance with the Standard Operating Procedures of South African National Parks.

Using the movement data from the collars, clusters of GPS positions where lions were stationary within a defined proximity (<100 m) for more than two consecutive GPS locations were identified as potential kill sites and investigated on foot to determine whether the lions had made a kill there or not (Tambling et al. 2010). Kills were identified and classified to prey species through forensic investigation of the cluster sites (i.e., the presence of carcass remains such as bones, hair, horns, or teeth), and the exact position of the kill was identified by the location of prey stomach contents when available (*n* = 125). When stomach contents were not found, the site of the carcass remains was used as the kill site (*n* = 81). Although the stomach contents location is often considered diagnostic of the actual kill site (Schaller 1972), in Addo, kill site characteristics where stomach contents are present are comparable to kill sites where only carcass remains are found (Davies et al. 2016). Moreover, the scale of our analysis (500 m buffers around camera traps) makes the exact location of a kill site less important. As no other large carnivores were present, we assumed that all lion feeding events were the result of lions making the kill, and not scavenged. The probability of a kill [*P*(Kill)] occurring in each camera trap buffer was then calculated as the proportion of kills (out of a total of 206 medium to large herbivore kills investigated over the study period) and used as a measure of mortality risk at each site.

We subsampled the lion location data to three readings per day for the female and one of the male lions that was collared for the longest time period. The second male was collared for a shorter period of time than the first male, namely from May 2012 to May 2013. We therefore used the data from the male with the longer collar history to represent the spatial location of the male coalition. Inspection of the collar data for the two males during the year that both were collared suggests that they spent up to ~99% of their time in close proximity to each other, enabling us to assume that their ranging behavior was the same (Appendix 1). Using the combined male and female datasets, we approximated the risk of lion encounter at a given location by an index proportional to the probability of lion presence at this location, following Valeix et al. (2009b). Consecutive 10% kernel isopleths were calculated using 0.6 times the reference smoothing factor *h*_ref_ for each individually collared lion. We used 0.6 of the *h*_ref_ in the analysis because *h*_*ref*_ tends to over‐smooth the data when locations are clumped and thus does not result in a range estimate that accurately identifies areas of known high use (Bertrand et al. 1996). We then approximated the probability of a lion being present by 0.1/(*A*_*i*_ *− A*_*i‐*1_); with *A*_*i*_ being the surface area of the isopleths *i* and 0.1, because 10% of all locations are located between two consecutive isopleths. Interpolated mean values for long‐term lion encounter risk were then extracted for each camera trap buffer. For these calculations, we used lion spatial data from when the lions were released in September 2011 until the GPS collar's batteries failed (June 2014 for the male lion and March 2014 for the female lion). We thus partitioned direct predation risk into two measures: lion encounter risk and mortality risk. Both of these measures were considered to represent long‐term, aggregated predation risk because they represented lion ranging and killing patterns over 34 months, much of which was prior to the camera trap survey.

In addition, we assessed short‐term predation risk as the risk of encountering a lion based on where the lions were during each individual camera trapping window. For this, we restricted the lion locations used in the analysis to those recorded during each camera trapping window for the cameras that were active while the lions were collared (*n* = 46). Following this, for each time period corresponding to an active camera, we divided the subsampled number of lion locations within each camera buffer by the total number of subsampled lion locations recorded across the entire study area during the same time period that the camera was active, thereby calculating the proportion of lion presence within each camera buffer over the time period the camera was active. For mortality risk, however, we used the same kill proportion data as before because the probability of a predator killing at a specific location is only partly related to the actual presence of the predator. Additional environmental factors also contribute to kill success (Davies et al. 2016), and it is not necessarily predator presence that makes a specific site dangerous for a prey species. Prey species might therefore still avoid sites where kills are likely to occur, even if a predator is not present at the time. In contrast, short‐term and long‐term predator encounter risk requires the predator to be in the area. Kill probability therefore reflects relative vulnerability, whereas predator encounter risk reflects the risk of encountering a predator and can be temporarily variable.

### Analysis

All statistical procedures were performed in R software, version 3.1.2 (R Development Core Team 2014). Collinearity between main effects for each dataset was assessed prior to all analyses using variance inflation factors (VIF) and Spearman rank correlation tests (Zuur et al. 2010). High levels of correlation were found between slope and terrain ruggedness (VRM), and slope and vegetation density for all datasets. Slope was therefore discarded from all analyses. All variables had VIF values below 3 (and most were below 2) in the final statistical models. VRM (multiplied by 10^3^), lion encounter risk (multiplied by 10^1^), and kill probability (multiplied by 10^1^) were rescaled in all models to ensure similar scales between predictor variables.

A candidate set of fourteen generalized linear mixed‐effects models with Poisson error distributions and log link functions was constructed to examine relationships between group abundance (calculated as the number of groups observed per 100 days of camera trap survey) of each herbivore species and vegetation density, terrain ruggedness (VRM), time of day, lion encounter risk, and mortality risk (kill probability). Two‐way interactions between habitat (vegetation density and terrain ruggedness) and predation risk variables (lion encounter risk and mortality risk), as well as between time of day and each other variable were included in the model sets to investigate whether herbivore species changed their spatial distributions in response to different forms of predation risk and between night and day (Tables A2.1, A2.2, A2.3, A2.4, A2.5, A2.6, A2.7, A2.8, A2.9, A2.10, A2.11, A2.12). This enabled us to test hypotheses about the relative importance of habitat and predation risk drivers of herbivore spatial distributions. Camera identity was set as a random effect in all models. Model selection was performed on the candidate models using Akaike information criteria corrected for sample size (AICc) and the model Akaike weights (AIC*w*_i_). Where there was close convergence between top models (cumulative Akaike weights between top models were <0.95), model averaging was implemented using the coefficients from the models that constituted a cumulative Akaike weight of 0.95 (Tables A2.3, A2.4, A2.6, and A2.8, Appendix 2) (Burnham and Anderson 2002). To visually assess the influence of each significant continuous predictor variable on the probability of a group being observed for each species, the median marginal probability of group presence was plotted as a function of the range of observed predictor variables. This was achieved by fixing the value of our variable of interest at 40 values across its observed range and, for each value, predicting the observed probability of group presence from the best candidate model while maintaining all other predictor variables (fixed and random) at their original input values (Elith et al. 2005). Such analyses were performed for each of the six prey species of interest as well as for long‐term and short‐term lion encounter risk.

## Results

**Figure 3:**
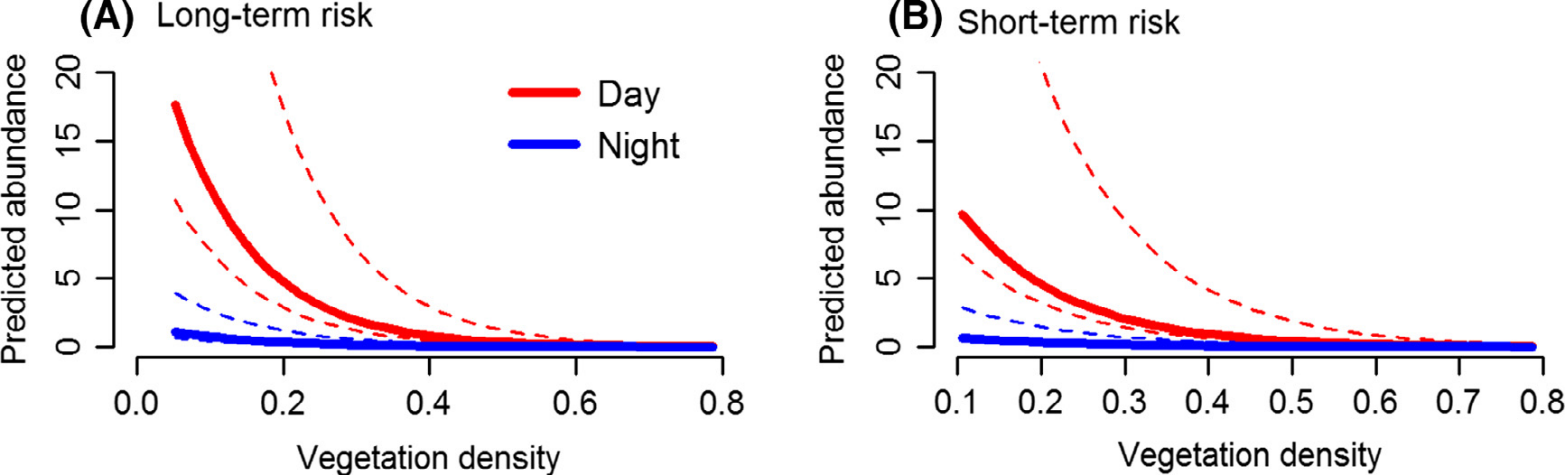
Relationships between ostrich group abundance, time of day, and vegetation density in the Nyathi section of the Addo Elephant National Park, South Africa when (A) lion encounter risk was averaged over 34 months and (B) lion encounter risk was restricted to the time each camera was active. Results are based on the most parsimonious model. Solid lines represent median values and dotted lines the 75% and 25% quantiles. The interaction between vegetation density and time of day was not significant but both main effects were (see Tables 1 and 2).

**Figure 4:**
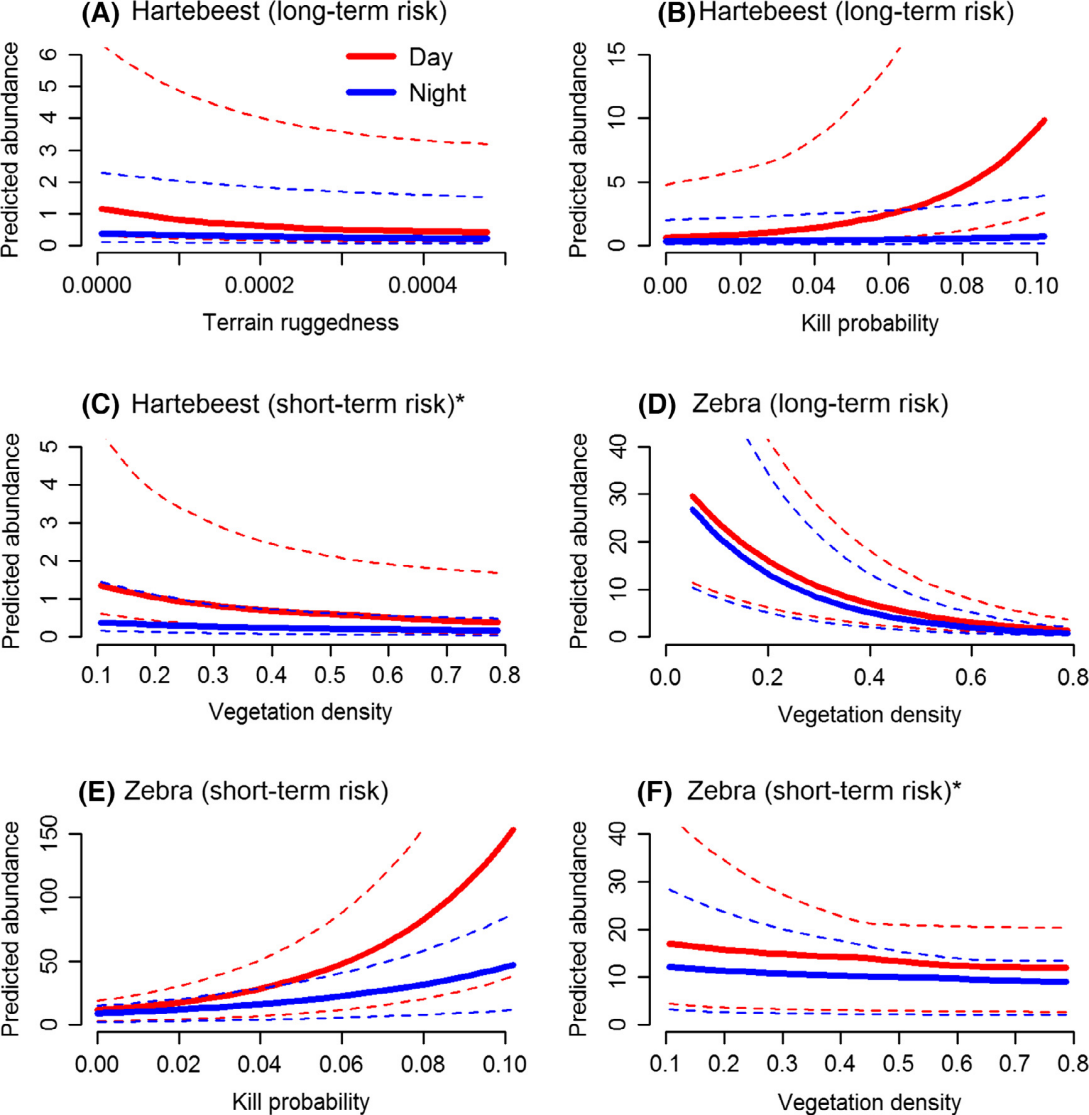
Relationships between group abundance and significant covariates for medium grazers (hartebeest and zebra) in the Nyathi section of the Addo Elephant National Park, South Africa when lion encounter risk was averaged over 34 months (A, B, and D) and restricted to the period each camera was active (C, E, and F). Results are based on the most parsimonious model for each species, or model averaging when there was close convergence between top models. Solid lines represent median values and dotted lines the 75 and 25% quantiles. Asterisks denote that the interaction term was not significant, only the main effects were (see Tables 1 and 2).

**Figure 5:**
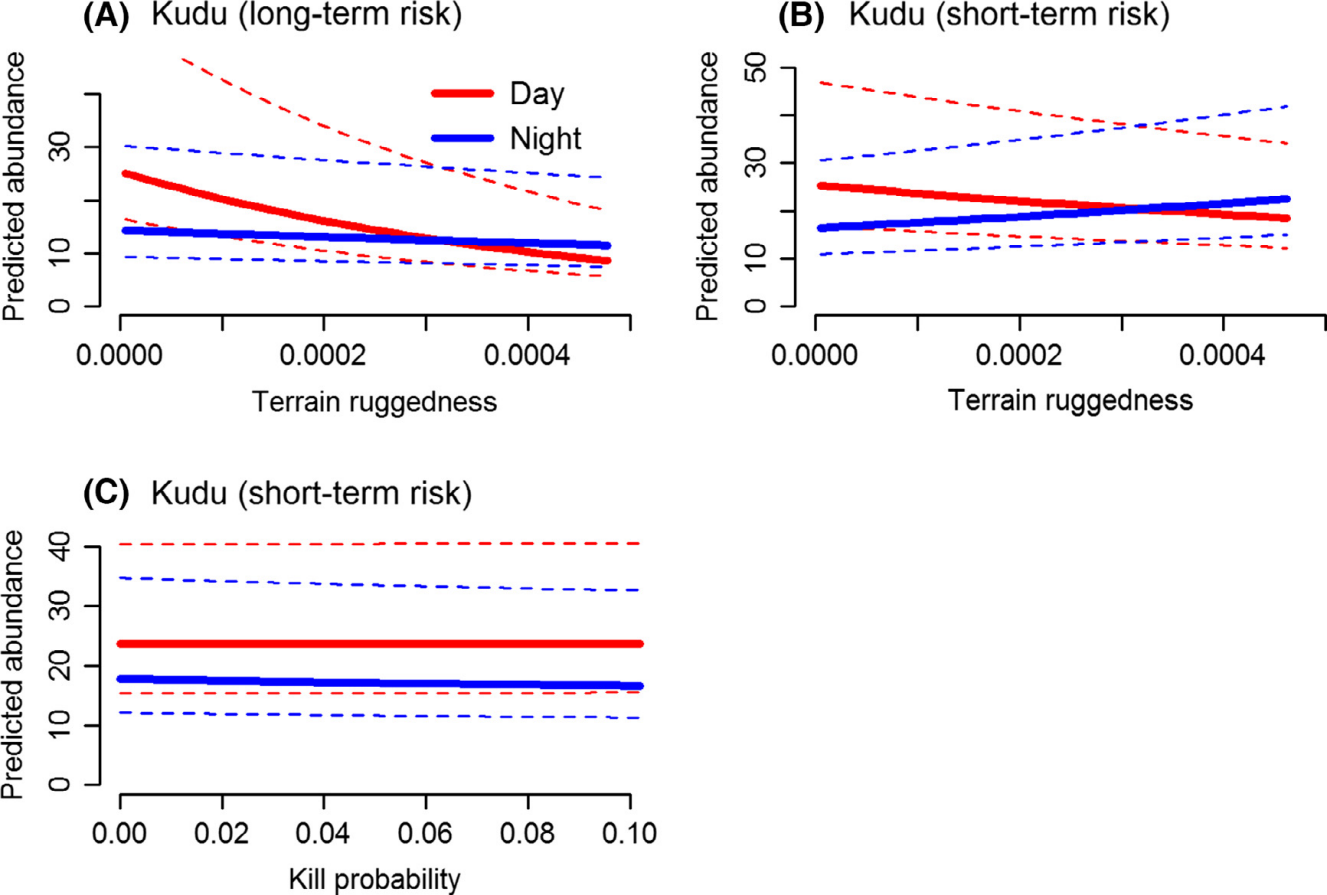
Relationships between kudu group abundance and significant covariates in the Nyathi section of the Addo Elephant National Park, South Africa when (A) lion encounter risk was averaged over 34 months and (B, C) lion encounter risk was restricted to the time each camera was active. Results are based on (A) the most parsimonious model or (B, C) model averaging. Solid lines represent median values and dotted lines the 75 and 25% quantiles. All main effects and their interactions were significant (see Tables 1 and 2).

**Figure 6:**
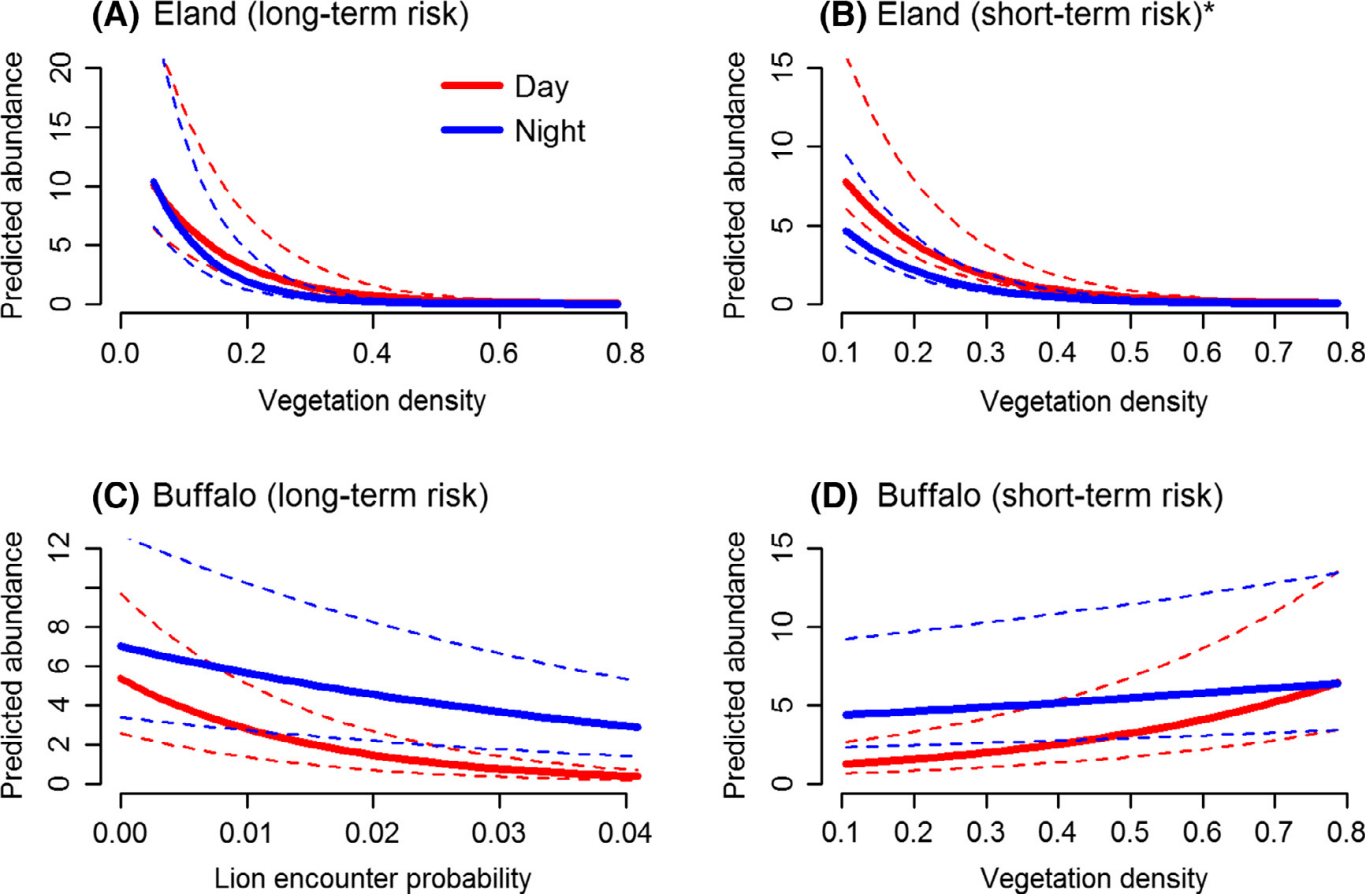
Relationships between group abundance and significant covariates for large prey species (eland and buffalo) in the Nyathi section of the Addo Elephant National Park, South Africa when lion encounter risk was averaged over 34 months (A, C) and restricted to the period each camera was active (B, D). Results are based on the most parsimonious model for each species. Solid lines represent median values and dotted lines the 75 and 25% quantiles. The asterisk at (B) denotes that the interaction was not significant, only the main effects were (see Tables 1 and 2).

For all herbivore species, apart from buffalo, environmental variables (time of day, vegetation density, and terrain ruggedness) were more important drivers of the distribution of prey groups than were direct measures of predation risk (lion encounter risk and mortality risk) when encounter risk was aggregated. Similarly, when encounter risk was restricted to short‐term risk, all species, apart from zebra, demonstrated stronger responses to environmental variables (Tables A2.1, A2.2, A2.3, A2.4, A2.5, A2.6, A2.7, A2.8, A2.9, A2.10, A2.11, A2.12, Appendix 2). Removing time of day from models resulted in considerable model fit deterioration (substantially higher AICc values – Tables A2.1, A2.2, A2.3, A2.4, A2.5, A2.6, A2.7, A2.8, A2.9, A2.10, A2.11, A2.12, Appendix 2), highlighting the importance of time of day for herbivore group observations. Vegetation density was included as a predictor in the best model for at least one dataset (aggregated or restricted encounter risk) for all species except kudu (Tables 1, 2, 3, 4, 5, 6, Figs 3, 4, 5, 6). In contrast, terrain ruggedness replaced vegetation density as the most important environmental predictor of group abundance for kudu (Table 4, Fig. 5). Our kill site investigations revealed that lions killed the six prey species of interest in differing frequencies, with ostrich comprising the highest proportion of kills and buffalo the least (Table 7).

**Table 1:** Model results for ostrich group abundance from camera trap surveys across the Nyathi section of Addo Elephant National Park, South Africa

Variable	Aggregated risk				Restricted risk			
	*β*	SE (*β*)	*Z*	*P*	*β*	SE (*β*)	*Z*	*P*
Time^a^	−2.85	0.34	−8.46	<0.001	−2.82	0.38	−7.53	<0.001
Vegetation	−8.90	1.66	−5.38	<0.001	−7.99	1.8	−4.44	<0.001
Time: vegetation	0.86	1.21	0.71	0.48	0.90	1.24	0.73	0.47

Compared to the reference category night.

**Table 2:** Model results for hartebeest group abundance from camera trap surveys across the Nyathi section of Addo Elephant National Park, South Africa. Model averaging was performed for both sets of encounter risk due to close convergence between top models (Tables A2.3 and A2.4, Appendix 2)

Variable	Aggregated risk				Restricted risk			
	*β*	SE (*β*)	*Z*	*P*	*β*	SE (*β*)	*Z*	*P*
Time^a^	−0.95	0.34	2.79	<0.01	−1.31	0.37	3.53	<0.001
Terrain	−6.06	3.07	1.95	0.05	−7.50	4.13	1.79	0.07
Time: terrain	2.68	0.99	2.67	<0.01	2.41	1.48	1.60	0.11
*P*(Kill)	3.81	1.93	1.95	0.05	3.61	2.07	1.72	0.09
Time: *P*(Kill)	−2.36	0.99	2.36	<0.05	−2.08	1.23	1.70	0.10
Vegetation	−4.45	1.51	2.93	<0.01	−4.31	1.76	2.42	<0.05
Time: vegetation	0.16	0.59	0.60	0.79	1.24	0.73	1.68	0.09

Compared to the reference category night.

**Table 3:** Model results for zebra group abundance from camera trap surveys across the Nyathi section of Addo Elephant National Park, South Africa. Model averaging was performed for short‐term encounter risk due to close convergence between top models (Table A2.6, Appendix 2)

Variable	Aggregated risk				Restricted risk			
	*β*	SE (*β*)	*Z*	*P*	*β*	SE (*β*)	*Z*	*P*
Time^a^	−0.06	0.001	−49.00	<0.001	−0.24	0.10	2.32	<0.05
Vegetation	−4.14	0.001	−3176.00	<0.001	−3.96	1.03	3.78	<0.001
Time: vegetation	−0.62	0.001	−479.00	<0.001	0.18	0.25	0.68	0.50
*P*(Kill)					14.10	6.40	2.17	<0.05
Time: *P*(Kill)					−4.81	1.36	3.49	<0.001

a Compared to the reference category night.

**Table 4:** Model results for kudu group abundance from camera trap surveys across the Nyathi section of Addo Elephant National Park, South Africa. Model averaging was performed for short‐term encounter risk due to close convergence between top models (Table A2.8, Appendix 2)

Variable	Aggregated risk				Restricted risk			
	*β*	SE (*β*)	*Z*	*P*	*β*	SE (*β*)	*Z*	*P*
Time^a^	−0.57	0.07	−8.82	<0.001	−0.44	0.12	3.5	<0.001
Terrain	−2.28	1.06	−2.16	<0.05	−0.79	1.08	0.72	0.47
Time: terrain	1.82	0.34	5.33	<0.001	1.57	0.43	3.58	<0.001
*P*(Kill)					0.05	0.6	0.08	0.94
Time: *P*(Kill)					−0.73	0.25	2.90	<0.01

a Compared to the reference category night.

**Table 5:** Model results for eland group abundance from camera trap surveys across the Nyathi section of Addo Elephant National Park, South Africa

Variable	Aggregated risk				Restricted risk			
	*β*	SE (*β*)	*Z*	*P*	*β*	SE (*β*)	*Z*	*P*
Time^a^	0.22	0.20	1.14	0.26	−0.42	0.22	−1.93	0.05
Vegetation	−7.80	1.63	−4.77	<0.001	−7.43	1.54	−4.82	<0.001
Time: vegetation	−3.61	0.80	−4.54	<0.001	−0.80	0.76	−1.05	0.30

a Compared to the reference category night.

**Table 6:** Model results for buffalo group abundance from camera trap surveys across the Nyathi section of Addo Elephant National Park, South Africa

Variable	Aggregated risk				Restricted risk			
	*β*	SE (*β*)	*Z*	*P*	*β*	SE (*β*)	*Z*	*P*
Time^a^	0.27	0.08	3.28	<0.01	1.45	0.19	7.69	<0.001
Encounter risk	−6.43	1.51	−4.25	<0.001				
Time: encounter	4.27	0.78	5.45	<0.001				
Vegetation					2.40	0.90	2.65	<0.01
Time: vegetation					−1.84	0.37	−4.96	<0.001

a Compared to the reference category night.

**Table 7:** Number and proportion of kills found for each prey species of interest as well as other species in the Nyathi section of the Addo Elephant National Park, South Africa. A total of 241 kills were found from forensic investigations of GPS clusters

Species	Number of kills	Proportion of kills
Ostrich	90	0.37
Hartebeest	48	0.20
Zebra	16	0.07
Kudu	21	0.09
Eland	29	0.12
Buffalo	2	0.01
Other species	35	0.15

Ostrich were observed more often during the day, and their presence at camera traps decreased significantly with an increase in vegetation density (Table 1, Fig. 3). There was no significant interaction between these two variables because very few ostrich groups were detected at night (6% of groups). These patterns remained similar for the aggregated and short‐term predation risk datasets (Table 1).

Observations of both medium grazers (hartebeest and zebra) decreased at night and when vegetation density was higher. The interaction between vegetation density and time of day was not significant for hartebeest group observations when lion encounter risk was either aggregated or restricted, indicating that for hartebeest, responses to vegetation density did not differ between the day and night. Similarly, for zebra, the interaction between vegetation density and time of day was not significant when encounter risk was restricted, and although statistically significant for aggregated encounter risk, predicted responses of zebra to vegetation density during the day and night suggest no biological significance (Fig. 4D). Assuming aggregated predation risk, hartebeest group abundance decreased with increasing ruggedness during the day, but not at night (Fig. 4A). Although a weaker predictor, mortality risk did feature for both species when model averaging was appropriate (both encounter risk datasets for hartebeest and restricted encounter risk for zebra), indicating that although a factor, it did not by itself explain species' responses. For both species, group observations increased with increasing mortality risk, particularly during the day (Fig. 4B,E). Habitat variables (vegetation density or terrain ruggedness) did not significantly interact with direct predation risk for either species.

In contrast to the other species, buffalo, the largest grazer, were observed more often in areas with high vegetation density and at night (Fig 6D) when lion encounter risk was restricted to the time each camera was active (Table 6). Furthermore, assuming aggregated encounter risk, fewer buffalo groups were observed when the risk of encountering a lion was high (Table 6, Fig. 6C). Responses during the day and night were superficially similar for both variables, with daytime responses being only slightly stronger (Fig. 6C,D). There were no significant interactions between habitat variables (vegetation density or terrain ruggedness) and direct predation risk for buffalo (Table 6).

Observations of eland, the only mixed feeder, decreased with time of day and vegetation density in similar ways to small (ostrich) and medium (hartebeest and zebra) grazers. However, this negative response to increasing vegetation density was stronger at night compared to the day (Table 5, Fig. 6A). No effects of direct predation risk (both lion encounter risk and mortality risk) were apparent for eland.

Kudu responded strongly to terrain ruggedness, and this response contrasted during the day and night. Kudu group observations decreased with increasing ruggedness during the day, but were either consistent at night (when lion encounter risk was aggregated, Fig. 5A) or increased with ruggedness (when encounter risk was restricted, Fig. 5B). Effects of direct predation risk (both lion encounter risk and mortality risk) were weaker for kudu, but mortality risk did feature when model averaging was appropriate (due to close convergence between top models – Table A2.4, Appendix 2). However, changes in group observations with kill probability were either not significant (main effect) or not biologically meaningful (interaction with time of day) (Table 4, Fig. 5C).

## Discussion

We found direct predation risk to have limited effects on the spatial distribution of a wide range of herbivore species varying in body size and foraging strategy. Instead, environmental characteristics were more important drivers, with time of day being a particularly important driver of herbivore group observations at a site. The relative importance of specific environmental attributes was related somewhat to herbivore body size and foraging strategy. Kudu was the only species not to respond to vegetation density, and buffalo was the only species observed more often at night and for which the number of groups increased with increasing vegetation density. We suggest that the limited effects of direct predation risk (lion encounter risk and mortality risk) are likely due to both the relatively low density of lions in the study area and their recent reintroduction following a 100‐year absence. Although this might appear as an “unnatural” scenario, it is an increasingly common one, with predator populations, including lions, declining across the globe (Ripple et al. 2014b; Bauer et al. 2015), and reintroductions of low numbers of predators elsewhere becoming more frequent (Hayward and Somers 2009). Moreover, predators, including lions, occur at naturally or artificially low densities in several ecosystems already (Packer et al. 2013; Bauer et al. 2015), and studies need to incorporate such areas before general conclusions about risk effects can be reached.

Our findings of limited direct risk effects corroborate recent findings from the Main Camp section of the Addo Elephant National Park, where recently (<2 years) reintroduced predators had limited influence on ungulate grouping behavior (Moll et al. 2016). Similarly, Scandinavian moose (*Alces alces*) displayed weak responses to the presence of recolonizing wolves (*Canis lupus*) (Nicholson et al. 2014). Although reintroduced predators have been shown to affect prey behavior elsewhere, including altering spatial distributions (Creel et al. 2005; Ripple and Beschta 2012), there is often a time lapse between reintroduction and discernible risk effects. Within Addo Main Camp, for example, buffalo took over 3 years to display behavioral adjustments in group size and habitat use to reintroduced lions, with considerable predation by lions on buffalo taking place before behavioral responses were initiated (Tambling et al. 2012). Although naïve prey are therefore more vulnerable to predation following reintroduction, they can learn to make behavioral adjustments and to recognize threats posed by predators within a single generation (Berger et al. 2001), meaning that initial vulnerability by prey need not curtail predator reintroduction efforts.

In addition to the recent time since reintroduction, the lion density in Nyathi was considerably lower than most regions where lions do not suffer high levels of human persecution (Packer et al. 2013) and might also be causal to the limited risk effects observed. Indeed, where direct lion associated risk effects have been detected, lion densities have generally been much higher than the 2.1 lions/100 km^2^ in our study site [Thaker et al. 2011 (5.8/100 km^2^), Tambling et al. 2012 (13.2/100 km^2^), Creel et al. 2014 (13.6/100 km^2^)]. Although direct predation risk effects were detected at similarly low lion densities in Hwange National Park (2.7 lions/100 km^2^), both lions and measured risk effects were largely concentrated around waterholes, resulting in locally high lion densities where herbivore behavioral responses were detected (Valeix et al. 2009a, 2010). Prey responses to predators and their reintroductions could therefore be stronger and develop faster when predator densities are high, but remain weak for longer periods, or permanently, when densities and subsequent predation rates are low (see Vucetich et al. 2011).

Although effects of direct predation risk were limited in our study, herbivore responses to the measured environmental variables likely reflect, at least partially, an indirect response to the threat of predation. Diel activity patterns reflect a trade‐off between background evolutionary processes and top‐down or bottom‐up driven activity bursts (Monterroso et al. 2013; Tambling et al. 2015). Therefore, the higher diurnal activity by vulnerable prey species suggests, at least partly, an indirect response to predation risk by nocturnal predators (i.e., lions), similar to observations in the adjacent Addo Main Camp (Tambling et al. 2015). Moreover, the significant interactions between time of day and vegetation density and/or terrain ruggedness are indicative of prey altering their behavior during risky times. Spatial adjustments by grazers and mixed feeders to both direct and indirect predation risk were generally stronger than those of browsers (kudu), demonstrated by larger effect sizes in measured responses. This contrasts with findings in African savannas where browsers moved into more open habitats when the risk of lion predation was high, whereas grazers remained in open areas regardless of predation risk (Valeix et al. 2009b). Lions are considered ambush predators in the sense that they make use of vegetation cover to stalk and surprise prey (Hopcraft et al. 2005). However, although this typical hunting behavior is used by lions in Addo, they do not make use of the densest vegetation available to them, but prefer to hunt within a fairly narrow band of vegetation density below the study site mean, partly because thicket attains densities substantially higher than savanna vegetation (Davies et al. 2016). Furthermore, based on the Nyathi lion utilization distribution, areas of high lion use were in relatively level, open areas, with areas of very dense vegetation being largely predator free (see also Tambling et al. 2013). Browsers in Nyathi might therefore be less vulnerable to lion predation than grazers because of their preference for denser vegetation and are subsequently less reliant on antipredator behavior. Alternatively, browsers could be constrained by their need for woody vegetation and be unable to move into open areas in the same way grazers do when predation risk is high. Although browsers are able to do this in savannas (Valeix et al. 2009b), open areas in Nyathi are remnants of old agricultural fields and contain comparatively little woody vegetation, as opposed to most savanna open areas that are interspersed with woody plant species. Contrasting responses to terrain ruggedness during the day and night by kudu do suggest that browsers display some response to indirect predation risk, moving into more rugged areas devoid of lions at night when the risk of predation is higher. However, even here effect sizes and changes in the number of groups observed were small compared to grazer responses.

All grazers and the mixed feeder, eland, were more abundant in open areas, which likely reflects responses to forage availability, although it could also be viewed as antipredator behavior for some species. Group observations of ostrich, the smallest grazer, were driven only by vegetation density and time of day. This avoidance of dense vegetation regardless of the time of day suggests that ostrich distribution represents a bottom‐up response (foraging constraints) rather than a predation risk response. This inability to respond to predation risk might be the main driver of ostrich being the most commonly consumed medium to large prey item by lions in Nyathi (Table 7). Both medium grazers, hartebeest and zebra, were also more abundant in open areas, probably driven by local forage availability. However, differing responses to vegetation density between day and night, with stronger differences for hartebeest, suggest that hartebeest are more sensitive to predation risk than zebra. Indeed, smaller and more vulnerable hartebeest comprise a greater proportion of lion kills in Nyathi compared with zebra. Furthermore, hartebeest displayed stronger avoidance of areas with a higher kill probability at night, reinforcing their comparatively greater sensitivity to predation risk. Although grazers would also be safe in very dense vegetation away from areas frequented by lions, they are unlikely to find sufficient forage in these habitats and are most likely forced into areas of overall higher lion predation risk (within the lion UD and where lions make kills). Furthermore, the encounter is only the first interaction between predator and prey, following which detection and escape become more important for prey (Heithaus et al. 2009; Wirsing et al. 2010). If a predator is detected earlier, such as in an open area, prey will have a better chance of escape and be able to do so more easily in open areas that contain fewer obstacles (Valeix et al. 2009b; Martin and Owen‐Smith 2016). Therefore, although these grazers reside in areas with a higher probability of lion encounter, fine‐scale behavioral responses once lions are encountered could be their primary antipredator behavior (see also Martin and Owen‐Smith 2016).

Large herbivores are generally less vulnerable to predation than small ones, partly due to smaller species being susceptible to a wider range of predators (Owen‐Smith and Mills 2008; Hopcraft et al. 2010). In Nyathi, this size‐dependent vulnerability is less important because lions were the only known resident predator of the prey species investigated and are capable of killing eland and buffalo, the two largest species. Buffalo were the only prey species to significantly avoid lion encounter risk. This behavior most likely enables them to be more active at night and to utilize denser vegetation. Prior to lion reintroduction in Addo Main Camp, buffalo were suggested to have been more nocturnal and to reside in areas of denser vegetation (Tambling et al. 2012). Similarly, prior to lion reintroduction in Nyathi, buffalo were active more often at night (Tambling et al. 2015). Our findings therefore show that buffalo have not altered their prelion behavior in Nyathi, most likely due to their avoidance of lions. Compared with buffalo, eland are killed by lions more often in Nyathi (Table 7) and remained in areas associated with lion presence. Although lions select hunting sites based on prey catchability rather than abundance (Hopcraft et al. 2005), at broad spatial scales they select areas with increased prey abundance and then select hunting areas within these where prey are easier to catch (Davidson et al. 2012). Accordingly, the high overlap between lion UD and where eland (and other common prey species) were often observed reflects lions selecting habitats with high prey availability. These prey species are then likely restricted to these areas by foraging and/or other constraints.

Although the spatial distributions of herbivores in Nyathi responded weakly to direct predation risk relative to environmental factors, we only assessed longer‐term predation risk. Variation in encounter risk over shorter, more immediate time periods (e.g., where predators and prey are at a given point in time) also influence antipredator behavior and the spatial distribution of prey (Creel et al. 2005; Valeix et al. 2009b; Martin and Owen‐Smith 2016). Such fine‐scale temporal avoidance might enable prey to preferentially use favored yet more risky habitat (under long‐term predation risk) when predators are absent, retreating to safer habitats when they are in the immediate vicinity. Such behavior could result in stronger risk effects than recorded here, but require finer‐scaled measurements of predator and prey distributions to be detected. Response strength based on prey traits might also be influenced by sexual dimorphism among prey species, with differential responses between sexes (Creel et al. 2005; Winnie and Creel 2007). Further investigation that considers sexual differences in body size and behavior (which has seldom been investigated for African species), in relation to the environment and predator reintroduction, will likely lead to refined understanding of trait‐based antipredator behavior.

Our study revealed that, in the thicket biome, the spatial distribution of the prey community following predator reintroduction is primarily determined by environmental factors, with prey species most likely focused on meeting forage requirements and avoiding proxies of predation risk rather than avoiding direct risk. However, it is difficult to decompose the causes of such limited risk effects because not only were the lions recently reintroduced, but they also persisted at a low density. If such limited risk effects were to persist beyond the time span of a single prey generation (Berger et al. 2001), this would suggest that the low predator density, rather than the recent reintroduction, was largely responsible for the limited direct risk effects. Further monitoring of the prey community is required for such testing and reinforces the need to conduct robust, long‐term monitoring of predator reintroductions in order to understand how predator–prey dynamics might change and to place such findings within the general context of predation risk effects (Tambling et al. 2012). As the array of studies investigating predation risk across differing environments and predator–prey communities expands, improved understanding of the role of predators in ecosystem functioning will emerge, facilitating better ecosystem management and anticipation of impacts when predators are either extirpated or reintroduced.

## Conflict of Interest

None declared.

## Figures and Tables

**Figure A1:**
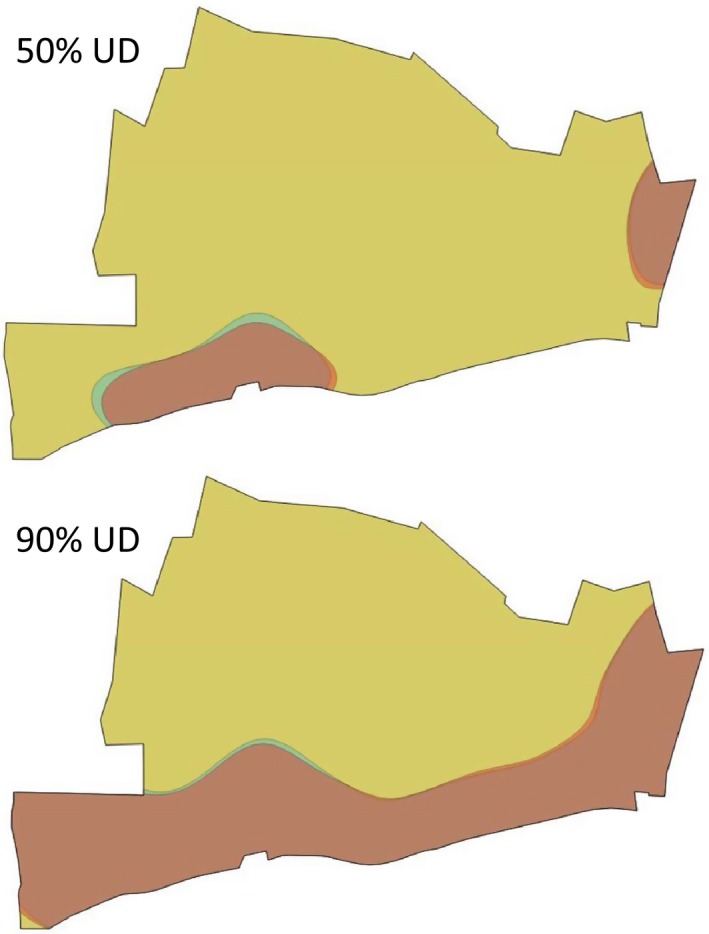
Map of the two male lion utilization distributions in Nyathi based on 50% and 90% of their core range during the time that both were collared with GPS units. The brown color represents the overlap in utilization distribution (UD) for the two male lions, whereas the slithers of blue and red represent the area of the UD for each male lion that is not covered by the other male lion in the coalition.

## Appendix 1

### Spatial relationships between the two male lions

The two male lions present in Nyathi at the time of the study were related and released together. On release in September 2011, only one of these male lions was collared. The second male was subsequently collared in May 2012, and this collar remained active until May 2013. Using the 12 months of overlapping telemetry data for the two lions, we calculated their 50% and 90% utilization distributions (UD) and found very high levels of spatial overlap (Table A1, Fig. A1). Furthermore, we investigated their locations in relation to each other based on the direct GPS locations. Because the download fixes of the two collars were not recorded simultaneously, there was a time lapse of about an hour between downloads for each individual. However, using GPS locations recorded within at least 66 min of each other, the lions were a mean distance of 390 m apart at each GPS download. If we assume that they moved a maximum of 4 km in an hour (roughly the time between downloads) away from their GPS location, they would have been together 99.39% of the time. When this maximum displacement was reduced 2 km in an hour, they would have been together 94.36% of the time, and 86.32% of the time if this distance was reduced further to only 1 km in an hour. We therefore assumed that the two male lions were together enough of the time to treat their ranging behavior as the same.

**Table A1:** Area, overlap, and relative difference in utilization distributions of the two male lions in Nyathi at the time of the study

Lion	Area (km^2^)	Overlap (%)	Difference (%)
50% utilization distribution			
Male 1	14.37	13.41	6.7
Male 2	13.89	13.41	3.5
90% utilization distribution			
Male 1	51.25	50.65	1.2
Male 2	51.41	50.65	1.5

## Appendix 2

**Table A2.1:** The regression model set used to describe the spatial distribution of ostrich groups in the Nyathi section of the Addo Elephant National Park, South Africa, when lion encounter risk was averaged over 34 months. Models are ranked according to Akaike information criterion (AICc) and Akaike weights (*w*_i_)

Form of regression model	AICc	∆AICc	*w*_i_
Time + Vegetation density + Time: vegetation density	404.97	0.00	0.99
Time + Ruggedness + Time: ruggedness	415.40	10.43	0.01
Time + Kill probability + Time: kill probability	425.81	20.84	0.00
Time + Encounter risk + Time: encounter risk	429.08	24.11	0.00
Time	436.25	31.28	0.00
Vegetation density + Ruggedness + Vegetation: ruggedness	901.25	496.28	0.00
Vegetation	904.90	499.93	0.00
Vegetation density + Encounter risk + Vegetation: encounter risk	907.93	502.97	0.00
Vegetation density + Kill probability + Vegetation: kill probability	908.53	503.56	0.00
Ruggedness	914.86	509.89	0.00
Ruggedness + Kill probability + Ruggedness: kill probability	917.95	510.93	0.00
Ruggedness + Encounter risk + Ruggedness: encounter risk	915.90	512.98	0.00
Kill probability	932.48	527.51	0.00
Encounter risk	933.88	528.91	0.00

**Table A2.2:** The regression model set used to describe the spatial distribution of ostrich groups in the Nyathi section of the Addo Elephant National Park, South Africa, when lion encounter risk was restricted to the time each camera was active. Models are ranked according to Akaike information criterion (AICc) and Akaike weights (*w*_i_)

Form of regression model	AICc	∆AICc	*w*_i_
Time + Vegetation density + Time: vegetation density	306.84	0.00	0.98
Time + Ruggedness + Time: ruggedness	314.69	7.85	0.02
Time + Kill probability + Time: kill probability	319.72	12.87	0.00
Time	325.87	19.03	0.00
Time + Encounter risk + Time: encounter risk	327.10	20.25	0.00
Vegetation	660.73	353.88	0.00
Vegetation density + Ruggedness + Vegetation: ruggedness	660.76	353.92	0.00
Vegetation density + Kill probability + Vegetation: kill probability	664.92	358.08	0.00
Vegetation density + Encounter risk + Vegetation: encounter risk	665.11	358.26	0.00
Ruggedness + Encounter risk + Ruggedness: encounter risk	668.81	361.97	0.00
Ruggedness	668.86	362.01	0.00
Ruggedness + Kill probability + Ruggedness: kill probability	672.15	365.30	0.00
Kill probability	678.72	371.87	0.00
Encounter risk	680.50	373.66	0.00

**Table A2.3:** The regression model set used to describe the spatial distribution of hartebeest groups in the Nyathi section of the Addo Elephant National Park, South Africa, when lion encounter risk was averaged over 34 months. Model averaging was conducted using the models with a cumulative Akaike weight >0.95 due to close convergence between top models. Models are ranked according to Akaike information criterion (AICc) and Akaike weights (*w*_i_)

Form of regression model	AICc	∆AICc	*w*_i_
Time + Ruggedness + Time: ruggedness	476.45	0.00	0.39
Time + Kill probability + Time: kill probability	476.78	0.33	0.33
Time + Vegetation density + Time: vegetation density	477.60	1.15	0.22
Time + Encounter risk + Time: encounter risk	481.07	4.62	0.04
Time	482.45	6.00	0.02
Vegetation	550.65	74.21	0.00
Vegetation density + Encounter risk + Vegetation: encounter risk	554.26	77.81	0.00
Vegetation density + Kill probability + Vegetation: kill probability	554.41	77.96	0.00
Vegetation density + Ruggedness + Vegetation: ruggedness	554.90	78.45	0.00
Kill probability	556.63	80.18	0.00
Ruggedness	556.76	80.31	0.00
Encounter risk	557.76	81.32	0.00
Ruggedness + Kill probability + Ruggedness: kill probability	559.02	82.57	0.00
Ruggedness + Encounter risk + Ruggedness: encounter risk	560.40	83.95	0.00

**Table A2.4:** The regression model set used to describe the spatial distribution of hartebeest groups in the Nyathi section of the Addo Elephant National Park, South Africa, when lion encounter risk was restricted to the time each camera was active. Model averaging was conducted using the models with a cumulative Akaike weight >0.95 due to close convergence between top models. Models are ranked according to Akaike information criterion (AICc) and Akaike weights (*w*_i_)

Form of regression model	AICc	∆AICc	*w*_i_
Time + Vegetation density + Time: vegetation density	317.68	0.00	0.53
Time + Kill probability + Time: kill probability	319.58	1.91	0.20
Time + Ruggedness + Time: ruggedness	320.33	2.66	0.14
Time	321.40	3.73	0.08
Time + Encounter risk + Time: encounter risk	322.51	4.83	0.05
Vegetation	395.26	77.59	0.00
Vegetation density + Ruggedness + Vegetation: ruggedness	397.30	79.62	0.00
Ruggedness	397.69	80.01	0.00
Kill probability	397.93	80.25	0.00
Encounter risk	399.08	81.41	0.00
Vegetation density + Kill probability + Vegetation: kill probability	399.11	81.43	0.00
Ruggedness + Kill probability + Ruggedness: kill probability	399.30	81.63	0.00
Vegetation density + Encounter risk + Vegetation: encounter risk	399.56	81.89	0.00
Ruggedness + Encounter risk + Ruggedness: encounter risk	399.97	82.30	0.00

**Table A2.5:** The regression model set used to describe the spatial distribution of zebra groups in the Nyathi section of the Addo Elephant National Park, South Africa, when lion encounter risk was averaged over 34 months. Models are ranked according to Akaike information criterion (AICc) and Akaike weights (*w*_i_)

Form of regression model	AICc	∆AICc	*w*_i_
Time + Vegetation density + Time: vegetation density	1121.98	0.00	0.88
Time + Kill probability + Time: kill probability	1126.02	4.04	0.12
Time + Ruggedness + Time: ruggedness	1141.32	19.34	0.00
Time	1149.17	27.20	0.00
Time + Encounter risk + Time: encounter risk	1150.04	28.07	0.00
Vegetation	1157.91	35.93	0.00
Vegetation density + Ruggedness + Vegetation: ruggedness	1158.18	36.20	0.00
Vegetation density + Encounter risk + Vegetation: encounter risk	1159.62	37.64	0.00
Vegetation density + Kill probability + Vegetation: kill probability	1161.68	39.70	0.00
Ruggedness	1169.93	47.95	0.00
Ruggedness + Kill probability + Ruggedness: kill probability	1170.05	48.08	0.00
Ruggedness + Encounter risk + Ruggedness: encounter risk	1173.86	51.88	0.00
Kill probability	1175.90	53.92	0.00
Encounter risk	1178.65	56.68	0.00

**Table A2.6:** The regression model set used to describe the spatial distribution of zebra groups in the Nyathi section of the Addo Elephant National Park, South Africa, when lion encounter risk was restricted to the time each camera was active. Model averaging was conducted using the models with a cumulative Akaike weight >0.95 due to close convergence between top models. Models are ranked according to Akaike information criterion (AICc) and Akaike weights (*w*_i_)

Form of regression model	AICc	∆AICc	*w*_i_
Time + Kill probability + Time: kill probability	759.37	0.00	0.71
Time + Vegetation density + Time: vegetation density	762.48	3.11	0.15
Time + Ruggedness + Time: ruggedness	762.83	3.46	0.13
Time + Encounter risk + Time: encounter risk	766.74	7.37	0.02
Time	772.21	12.84	0.00
Vegetation density + Ruggedness + Vegetation: ruggedness	793.63	34.26	0.00
Vegetation	799.31	39.94	0.00
Ruggedness + Kill probability + Ruggedness: kill probability	801.00	41.63	0.00
Ruggedness	801.35	41.98	0.00
Vegetation density + Encounter risk + Vegetation: encounter risk	802.15	42.78	0.00
Vegetation density + Kill probability + Vegetation: kill probability	803.48	44.11	0.00
Ruggedness + Encounter risk + Ruggedness: encounter risk	804.00	44.63	0.00
Kill probability	809.31	49.94	0.00
Encounter risk	811.80	52.43	0.00

**Table A2.7:** The regression model set used to describe the spatial distribution of kudu groups in the Nyathi section of the Addo Elephant National Park, South Africa, when lion encounter risk was averaged over 34 months. Models are ranked according to Akaike information criterion (AICc) and Akaike weights (*w*_i_)

Form of regression model	AICc	∆AICc	*w*_i_
Time + Ruggedness + Time: ruggedness	1452.01	0.00	1.00
Time + Kill probability + Time: kill probability	1464.03	12.02	0.00
Time + Vegetation density + Time: vegetation density	1476.21	24.20	0.00
Time	4178.02	26.01	0.00
Time + Encounter risk + Time: encounter risk	1479.01	27.00	0.00
Vegetation density + Ruggedness + Vegetation: ruggedness	1531.84	79.83	0.00
Vegetation density + Encounter risk + Vegetation: encounter risk	1535.24	83.23	0.00
Vegetation	1536.80	84.79	0.00
Vegetation density + Kill probability + Vegetation: kill probability	1537.28	85.27	0.00
Ruggedness	1539.15	87.14	0.00
Ruggedness + Kill probability + Ruggedness: kill probability	1539.87	87.86	0.00
Ruggedness + Encounter risk + Ruggedness: encounter risk	1540.48	88.47	0.00
Kill probability	1540.54	88.53	0.00
Encounter risk	1540.77	88.76	0.00

**Table A2.8:** The regression model set used to describe the spatial distribution of kudu groups in the Nyathi section of the Addo Elephant National Park, South Africa, when lion encounter risk was restricted to the time each camera was active. Model averaging was conducted using the models with a cumulative Akaike weight >0.95 due to close convergence between top models. Models are ranked according to Akaike information criterion (AICc) and Akaike weights (*w*_i_)

Form of regression model	AICc	∆AICc	*w*_i_
Time + Ruggedness + Time: ruggedness	1123.10	0.00	0.79
Time + Kill probability + Time: kill probability	1127.08	3.98	0.11
Time + Vegetation density + Time: vegetation density	1127.83	4.73	0.07
Time + Encounter risk + Time: encounter risk	1131.51	8.41	0.01
Time	1131.75	8.65	0.00
Vegetation density + Ruggedness + Vegetation: ruggedness	1156.75	33.64	0.00
Vegetation	1162.46	39.35	0.00
Encounter risk	1165.56	42.46	0.00
Vegetation density + Kill probability + Vegetation: kill probability	1166.49	43.39	0.00
Vegetation density + Encounter risk + Vegetation: encounter risk	1166.59	43.49	0.00
Kill probability	1166.95	43.85	0.00
Ruggedness	1167.11	44.01	0.00
Ruggedness + Kill probability + Ruggedness: kill probability	1169.16	46.06	0.00
Ruggedness + Encounter risk + Ruggedness: encounter risk	1169.41	46.31	0.00

**Table A2.9:** The regression model set used to describe the spatial distribution of eland groups in the Nyathi section of the Addo Elephant National Park, South Africa, when lion encounter risk was averaged over 34 months. Models are ranked according to Akaike information criterion (AICc) and Akaike weights (*w*_i_)

Form of regression model	AICc	∆AICc	*w*_i_
Time + Vegetation density + Time: vegetation density	596.69	0.00	1.00
Time + Ruggedness + Time: ruggedness	619.26	22.57	0.00
Time + Kill probability + Time: kill probability	625.22	28.53	0.00
Time + Encounter risk + Time: encounter risk	631.19	34.50	0.00
Vegetation density + Ruggedness + Vegetation: ruggedness	650.47	53.78	0.00
Time	651.28	54.59	0.00
Ruggedness + Kill probability + Ruggedness: kill probability	651.95	55.26	0.00
Ruggedness + Encounter risk + Ruggedness: encounter risk	657.66	60.97	0.00
Ruggedness	661.45	64.76	0.00
Vegetation density + Kill probability + Vegetation: kill probability	662.14	65.45	0.00
Vegetation	662.94	66.25	0.00
Vegetation density + Encounter risk + Vegetation: encounter risk	663.14	66.45	0.00
Encounter risk	679.31	82.62	0.00
Kill probability	683.52	86.83	0.00

**Table A2.10:** The regression model set used to describe the spatial distribution of eland groups in the Nyathi section of the Addo Elephant National Park, South Africa, when lion encounter risk was restricted to the time each camera was active. Although there was close convergence between top models, model averaging was not implemented because only the top model was included in the model set when cumulative Akaike weights were > 0.95. Models are ranked according to Akaike information criterion (AICc) and Akaike weights (*w*_i_)

Form of regression model	AICc	∆AICc	*w*_i_
Time + Vegetation density + Time: vegetation density	372.17	0.00	0.66
Time + Ruggedness + Time: ruggedness	373.49	1.32	0.34
Time + Encounter risk + Time: encounter risk	383.78	11.61	0.00
Time + Kill probability + Time: kill probability	395.24	23.08	0.00
Vegetation density + Ruggedness + Vegetation: ruggedness	395.33	23.16	0.00
Ruggedness + Kill probability + Ruggedness: kill probability	397.01	24.85	0.00
Vegetation	397.30	25.13	0.00
Time	399.62	27.46	0.00
Vegetation density + Kill probability + Vegetation: kill probability	400.05	27.88	0.00
Vegetation density + Encounter risk + Vegetation: encounter risk	401.03	28.86	0.00
Ruggedness	408.44	36.28	0.00
Ruggedness + Encounter risk + Ruggedness: encounter risk	409.00	36.83	0.00
Kill probability	419.48	47.31	0.00
Encounter risk	421.29	49.12	0.00

**Table A2.11:** The regression model set used to describe the spatial distribution of buffalo groups in the Nyathi section of the Addo Elephant National Park, South Africa, when lion encounter risk was averaged over 34 months. Models are ranked according to Akaike information criterion (AICc) and Akaike weights (*w*_i_)

Form of regression model	AICc	∆AICc	*w*_i_
Time + Encounter risk + Time: encounter risk	835.17	0.00	0.87
Time + Vegetation density + Time: vegetation density	838.98	3.80	0.13
Time + Ruggedness + Time: ruggedness	857.87	22.70	0.00
Time + Kill probability + Time: kill probability	864.19	29.02	0.00
Time	870.76	35.59	0.00
Ruggedness + Kill probability + Ruggedness: kill probability	921.95	86.78	0.00
Ruggedness + Encounter risk + Ruggedness: encounter risk	922.03	86.86	0.00
Ruggedness	923.29	88.11	0.00
Vegetation density + Ruggedness + Vegetation: ruggedness	926.16	90.99	0.00
Kill probability	932.22	97.04	0.00
Encounter risk	934.64	99.46	0.00
Vegetation density + Kill probability + Vegetation: kill probability	935.99	100.82	0.00
Vegetation density + Encounter risk + Vegetation: encounter risk	937.78	102.61	0.00
Vegetation	938.01	102.84	0.00

**Table A2.12:** The regression model set used to describe the spatial distribution of buffalo groups in the Nyathi section of the Addo Elephant National Park, South Africa, when lion encounter risk was restricted to the time each camera was active. Models are ranked according to Akaike information criterion (AICc) and Akaike weights (*w*_i_)

Form of regression model	AICc	∆AICc	*w*_i_
Time + Vegetation density + Time: vegetation density	625.92	0.00	0.91
Time + Ruggedness + Time: ruggedness	630.69	4.77	0.08
Time + Encounter risk + Time: encounter risk	634.92	9.00	0.01
Time + Kill probability + Time: kill probability	645.52	19.60	0.00
Time	648.24	22.32	0.00
Vegetation density + Ruggedness + Vegetation: ruggedness	700.64	74.72	0.00
Ruggedness	700.70	74.78	0.00
Ruggedness + Encounter risk + Ruggedness: encounter risk	701.05	75.13	0.00
Ruggedness + Kill probability + Ruggedness: kill probability	701.29	75.37	0.00
Kill probability	707.77	81.85	0.00
Encounter risk	710.95	85.03	0.00
Vegetation	711.76	85.84	0.00
Vegetation density + Kill probability + Vegetation: kill probability	712.08	86.16	0.00
Vegetation density + Encounter risk + Vegetation: encounter risk	714.10	88.18	0.00
